# A Reappraisal of Ventilatory Thresholds in Wheelchair Athletes With a Spinal Cord Injury: Do They Really Exist?

**DOI:** 10.3389/fphys.2021.719341

**Published:** 2021-11-26

**Authors:** Julia Kathrin Baumgart, Gertjan Ettema, Katy E. Griggs, Victoria Louise Goosey-Tolfrey, Christof Andreas Leicht

**Affiliations:** ^1^Department of Neuromedicine and Movement Science, Centre for Elite Sports Research, Norwegian University of Science and Technology, Trondheim, Norway; ^2^The Peter Harrison Centre for Disability Sport, School of Sport, Exercise and Health Sciences, Loughborough University, Loughborough, United Kingdom; ^3^Department of Engineering, School of Science and Technology, Nottingham Trent University, Nottingham, United Kingdom

**Keywords:** threshold, respiratory, gas exchange, tetraplegia, paraplegia, paralympic

## Abstract

The ventilatory threshold (VT) separates low- from moderate-intensity exercise, the respiratory compensation point (RCP) moderate- from high-intensity exercise. Both concepts assume breakpoints in respiratory data. However, the objective determination of the VT and RCP using breakpoint models during upper-body modality exercise in wheelchair athletes with spinal cord injury (SCI) has received little attention. Therefore, the aim of this study was to compare the fit of breakpoint models (i.e., two linear regression lines) with continuous no-breakpoint models (i.e., exponential curve/second-order polynomial) to respiratory data obtained during a graded wheelchair exercise test to exhaustion. These fits were compared employing adjusted R^2^, and blocked bootstrapping was used to derive estimates of a median and 95% confidence intervals (CI). V̇O_2_-V̇CO_2_ and V̇E/V̇O_2_-time data were assessed for the determination of the VT, and V̇CO_2_-V̇E and V̇E/V̇CO_2_-time data for the determination of the RCP. Data of 9 wheelchair athletes with tetraplegia and 8 with paraplegia were evaluated. On an overall group-level, there was an overlap in the adjusted R^2^ median ± 95% CI between the breakpoint and the no-breakpoint models for determining the VT (V̇O_2_-V̇CO_2_: 0.991 ± 0.003 vs. 0.990 ± 0.003; V̇E/V̇O_2_-time: 0.792 ± 0.101 vs. 0.782 ± 0.104, respectively) and RCP (V̇E-V̇CO_2_: 0.984 ± 0.004 vs. 0.984 ± 0.004; V̇E/V̇CO_2_-time: 0.729 ± 0.064 vs. 0.691 ± 0.063, respectively), indicating similar model fit. We offer two lines of reasoning: (1) breakpoints in these respiratory data exist but are too subtle to result in a significant difference in adjusted R^2^ between the investigated breakpoint and no-breakpoint models; (2) breakpoints do not exist, as has been argued previously.

## Introduction

In order to optimize endurance performance, athletes with an impairment often structure their training according to exercise domains, described by low-, moderate-, and high-intensity zones (Bhambhani et al., 1995; Coutts and McKenzie, 1995; Zeller et al., 2017). These exercise domains are distinguished using thresholds with a variety of methods that are based on either critical power (Vanhatalo et al., 2011; Poole et al., 2016), blood lactate (Faude et al., 2009), and/or respiratory data (Meyer et al., 2005). For the respiratory data, the gas exchange threshold (GET) separates low- from moderate-intensity exercise and the respiratory compensation point (RCP) separates moderate- from high-intensity exercise. Most threshold determination methods that are based on respiratory data have an *a priori* assumption of the presence of a breakpoint, defined as “a place where an interruption or change occurs” (Definition Breakpoint, 2017) and are commonly based on subjective determination of these breakpoints (Aunola and Rusko, 1984; Gladden et al., 1985; Bhambhani et al., 1993; Holland et al., 1994; Leicht et al., 2014; Kouwijzer et al., 2019). The GET is determined by a breakpoint in the V̇O_2_-V̇CO_2_ (V-slope method) (Beaver et al., 1986) or V̇E/V̇O_2_-time relationship (ventilatory equivalent method) (Reinhard et al., 1979). The RCP marks the exercise intensity beyond which any attempt to maintain homeostasis fails and is marked by hyperventilation and an exponential increase in V̇CO_2_. The RCP is determined by a breakpoint in the V̇E/V̇CO_2_-time (ventilatory equivalent method) (Reinhard et al., 1979) or V̇CO_2_-V̇E relationship (Beaver et al., 1986). Whilst there is an abundance of research focusing on the use of these methods in able-bodied athletes during lower- or whole-body exercise (Seiler and Kjerland, 2006; Seiler and Tønnessen, 2009; Pettitt et al., 2013), there is a lack of studies identifying these breakpoints to distinguish exercise domains in wheelchair athletes during upper-body exercise.

The presence of breakpoints in gas exchange data is a debated topic (Myers and Ashley, 1997; Hopker et al., 2011). Hopker et al. (2011) suggests that the V̇O_2_, V̇CO_2_ and V̇E response to an increase in exercise intensity might be curvilinear rather than characterized by clear breakpoints. Alternatively, assuming breakpoints exist (Meyer et al., 2005), their determination during upper-body exercise might be further complicated as the absolute maximum values of respiratory variables, and hence their range, are smaller compared to lower-body or whole-body exercise primarily due to less active muscle mass. This effect may be even more noticeable in athletes with a spinal cord injury (SCI) for whom respiratory responses are highly variable (Gee et al., 2019; Panza et al., 2019; Panza and Guccione, 2020) and the range of respiratory responses is further limited due to larger reductions in active muscle mass (Coutts et al., 1983). Indeed, respiratory dysfunction is pronounced in athletes with a tetraplegia (TETRA; cervical SCI), who—depending on the injury level—have intercostal and abdominal muscle paralysis and impaired diaphragm function (Theisen, 2012). In addition, the lower peak blood lactate concentrations in TETRA may further reduce the extent of metabolically driven hyperventilation, which is also supported by lower peak ventilation rates in TETRA indicative of altered respiratory responses *during* exercise (Leicht et al., 2014). As such, breakpoints in respiratory data may even be less distinct in TETRA. So far, whether breakpoints exist in the respiratory data of athletes with an impairment has only been investigated in a group of Para ice hockey players with different disabilities of the lower limbs and/or lower trunk (Baumgart et al., 2018). In this study, continuous no-breakpoint models fit the respiratory data better compared with breakpoint models (Baumgart et al., 2018), supporting the notion that breakpoints may not exist for this population and/or modality. However, the test protocol employed in this study was not a continuous graded test to exhaustion as usually used for the analysis of ventilatory thresholds (Meyer et al., 2005), but intermittent with short breaks between each of the 5-min stages to allow for blood sampling. The data may hence not be directly comparable to that obtained during continuous graded exercise tests, for which more comparative values exist. The present study addresses this limitation and further extends this research by investigating the possible effect of the level of SCI on the presence or absence of clear breakpoints during upper-body exercise, by focusing on wheelchair athletes with TETRA and paraplegia (PARA).

The aim of this study was therefore to investigate whether distinct breakpoints exist in the respiratory data obtained during a continuous graded exercise test to exhaustion in TETRA and PARA by using automated model fitting procedures.

## Materials and Methods

### Participants

Seventeen international wheelchair court sport players split into two groups according to SCI level—9 TETRA (1 woman, 8 men) and 8 PARA (1 woman, 7 men)—volunteered for this study (Table 1). All athletes used a wheelchair for daily ambulation. Most athletes self-reported to have a sensory- and motor-complete SCI. 2 TETRA had a sensory-incomplete and 1 a sensory- and motor-incomplete injury. 3 PARA had a motor-incomplete and 1 a sensory-incomplete injury. The study was approved by the Ethical Committee of Loughborough University and conducted in accordance with the Declaration of Helsinki. All participants provided written informed consent prior to the data collection and further completed separate health, training and impairment questionnaires. Note that the data were obtained as part of a previously published study by Leicht et al. (2014). However, we removed the data of one PARA and one TETRA due to incomplete data that would have been required for the model fitting approach chosen in the present study.

**TABLE 1:** Age, anthropometric characteristics as well as peak cardio-respiratory parameters of the 17 wheelchair athletes participating in this study.

**Parameter**	**TETRA (*n* = 9)**	**PARA (*n* = 8)**
Age (years)	28.5 ± 4.2	25.6 ± 6.6
Body mass (kg)	67.0 ± 12.1	63.9 ± 11.0
Body height (cm)	186 ± 8	173 ± 8^a^
Level of spinal cord injury	C5–C6/7	T4–L4
Absolute V̇O_2peak_ (L⋅min^–1^)	1.58 ± 0.32	2.09 ± 0.56^b^
Body-mass adjusted V̇O_2peak_ (mL⋅kg^–1^⋅min^–1^)	23.9 ± 4.9	33.7 ± 10.6^b^
Peak minute ventilation (L⋅min^–1^)	66.3 ± 11.8	87.1 ± 25.9^a^
Peak heart rate (beats⋅min^–1^)	131 ± 8	180 ± 13^a^
Peak blood lactate concentration (mmol⋅L^–1^)	5.15 ± 1.09	6.50 ± 1.65^b^
Resting blood lactate concentration (mmol⋅L^–1^)	0.85 ± 0.28	0.92 ± 0.15

*Note that the cardio-respiratory parameters were calculated as described in Leicht et al. (2014).*

*C, cervical injury level; TETRA, wheelchair athletes with tetraplegia; PARA, wheelchair athletes with paraplegia; T, thoracic injury level; L, lumbar injury level.*

*^a^Significantly different to TETRA at an α level of 0.05.*

*^b^Trend towards significant difference to TETRA at an a level of >0.05 but <0.1.*

### Experimental Design and Data Collection

For a detailed description of the experimental design, see Leicht et al. (2014). In brief, participants performed a continuous graded exercise test to exhaustion in their sports wheelchair on a motorized treadmill (HP Cosmos, Traunstein, Germany) at a constant 1.0% gradient. Wheelchairs were secured to a safety rail located on the side of the treadmill that kept the wheelchair centered but allowed free back and forward movement within the treadmill dimension. The starting speed of the test was set between 1.2 and 2.0 m⋅s^–1^ with 0.2–0.4 m⋅s^–1^ increments every 3 min, which were individually adjusted based on the differences in impairment and fitness levels. The test was terminated when participants were unable to maintain the speed of the treadmill, touching the “backstop” of the safety rail for the third time. Respiratory data (V̇O_2_, V̇CO_2_, V̇E) were recorded continuously using an online gas analysis system in breath-by-breath mode (Meta-Lyzer 3B, Cortex Biophysik GmbH, Leipzig, Germany).

### Data Processing

The data of TETRA and PARA was previously analyzed in the study of Leicht et al. (2014), where blood lactate and respiratory thresholds between TETRA and PARA were determined by expert opinion and then compared. This is a subjective approach, which we extend on in the current study by determining thresholds using objective, automated procedures.

In the current study, a breakpoint model (i.e., two regression lines, Equation 1) and a continuous curvilinear (no-breakpoint) model (i.e., an exponential curve, Equation 2) were fitted to the V̇O_2_-V̇CO_2_ (identification of GET) and the V̇CO_2_-V̇E (identification of RCP) data. For the V̇E/V̇O_2_-time (identification of GET) and V̇E/V̇CO_2_-time (identification of RCP) data, the continuous curvilinear (no-breakpoint) model was a second order polynomial (Equation 3). We considered the identification of the GET and RCP by the ventilatory equivalent method valid if the breakpoint in the V̇E/V̇O_2_-time occurred before the breakpoint in the V̇E/V̇CO_2_-time data (Powers et al., 1984; Gaskill et al., 2001). Matlab (R2021a; Mathworks Inc., Natick, MA) was used to perform the model fitting procedures. The function *slmengine* of the Shape Language Modeling (SLM) toolkit (D’Errico, 2021) was used to fit two regression lines to the data, including the function *fmincon* with the interior-point algorithm to find the best fitting model. A custom-made function was written to fit the exponential curve to the data, and the function *fminsearch* using the Nelder-Mead approach (Nelder and Mead, 1965) was used to determine the best fit with a maximum of 40,000 evaluations and 1,500 iterations. The function *polyfit* was used to fit the second-order polynomial to the data.


(1) $$y=\left\{ \begin{array}{c} a_{1}+b_{1}x,t<k\\ a_{2}+b_{2}x,t\geq k \end{array}\right.$$

(2) $$y=a+c\cdot exp\left(\frac{x}{d}\right)$$

(3) $$y=a+e\cdot x+f\cdot x^{2}$$

where *y* is the variable of interest, *a* the y-axis offset, *b* to *f* the slope coefficients, and *k* represents the intersection point of the first and the second regression lines of the piecewise function. Prior to fitting the breakpoint and no-breakpoint models to the breath-by-breath data an outlier detection was performed. Data points were considered outliers and removed if they exceeded 3 standard deviations from a third-order polynomial that was fitted to the data of each individual participant (removal of mean percentage ± SD of data points in V̇O_2_-V̇CO_2_ data: 1.4 ± 0.6%, V̇E/V̇O_2_-time data: 1.2 ± 0.4%, V̇E/V̇CO_2_-time data: 1.9 ± 0.8%, V̇CO_2_-V̇E data: 1.4 ± 0.6%).

### Statistics

The participant characteristics provided in Table 1 were compared by employing independent samples *T*-tests. An α level of 0.05 was used to indicate statistical significance. The adjusted R^2^ (Equation 4) was used to compare the fit of the breakpoint model (Equation 1) with the fit of the continuous curvilinear model (Equations 2 and 3) for each individual participant.


(4) $$AdjustedR^{2}=1-\frac{\left(1-R^{2}\right)\left(N-1\right)}{N-p-1}$$

where *p* is the number of coefficients in the model and *N* the number of data points. The adjusted R^2^ was used to account for the different number of coefficients, with four coefficients for the two regression lines, and three coefficients for the exponential curve and second-order polynomial. The overlapping blocked bootstrap technique with 10-s intervals was chosen to account for dependency in the data (Davison and Hinkley, 1997; Bühlmann, 2002), and used to attain one hundred random samples of the underlying respiratory data for each participant. An adjusted R^2^ value was then obtained for the fit of each model to each of the resampled V̇O_2_-V̇CO_2_, V̇E/V̇O_2_-time, V̇CO_2_-V̇E, and V̇E/V̇CO_2_-time data. This resulted in 100 adjusted R^2^ values for each model for every individual participant. The difference between the adjusted R^2^ values, and thus fit of the breakpoint model compared with the non-breakpoint model, for each individual and each of the approaches (i.e., two approaches for the GET and two approaches for the RCP) was assessed by comparing notched boxplots. The notches are 95% CIs that are constructed around the median (see Supplementary Figure 1). No overlap in the notches indicates 95% confidence of a difference between medians (Chambers et al., 1983). Our rationale was that a better fit of the continuous curvilinear model (no-breakpoint model) as compared with the two regression lines (breakpoint model), would challenge the presence of a distinct breakpoint. Notched boxplots were also used to assess whether the adjusted R^2^ differed between breakpoint and no-breakpoint models with the data of TETRA and PARA pooled, as well as to investigate whether there was a difference in model fit within and between those two groups.

## Results

**FIGURE 1:**
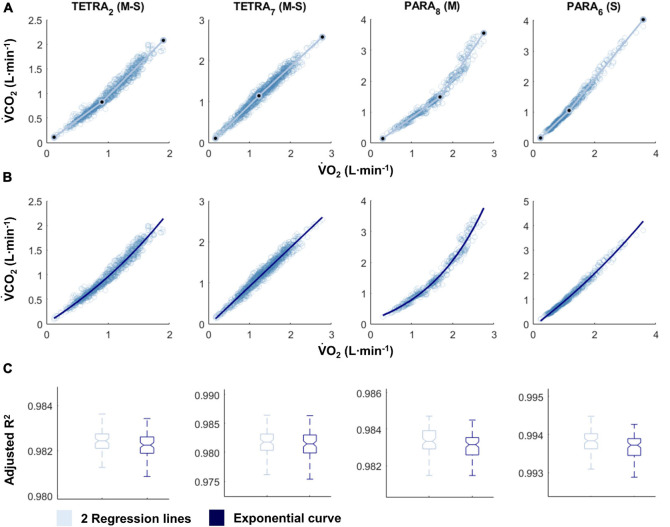
V̇O_2_-V̇CO_2_ plots fitted with the two linear regression lines (breakpoint model, V-slope method; **A**) and an exponential curve (no-breakpoint model; **B**) for example athletes with tetraplegia and paraplegia who exhibit the most and least distinct breakpoint within their respective groups (TETRA_2_, TETRA_7_; PARA_8_, PARA_6_; participant number in subscript). Notched boxplots are presented to compare the adjusted R^2^ values between the breakpoint and the no-breakpoint model for each participant **(C)**. Figures for all individual participants are provided in Supplementary Figure 2. V̇O_2,_ Oxygen uptake; V̇CO_2_, carbon dioxide production; TETRA, wheelchair athlete with tetraplegia; PARA, wheelchair athlete with paraplegia, M, motor complete injury; S, sensory complete injury.

**FIGURE 2:**
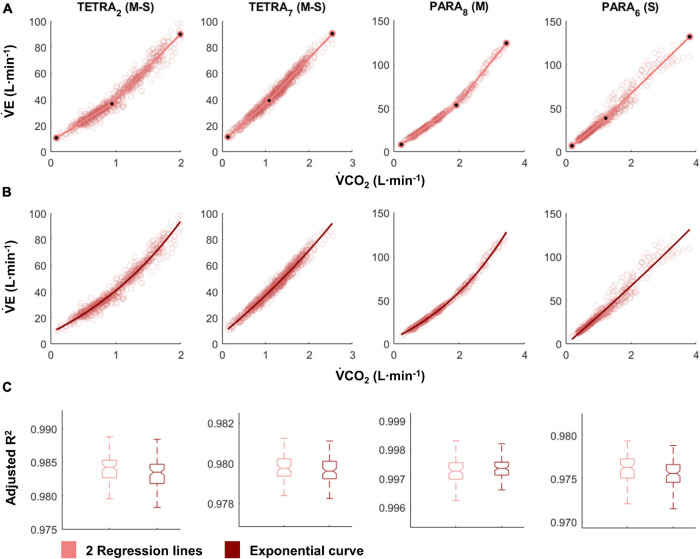
V̇CO_2_-V̇E plots fitted with the two linear regression lines (breakpoint model, respiratory compensation point method; **A**) and an exponential curve (no-breakpoint model; **B**) for example athletes with tetraplegia and paraplegia who exhibit the most and least distinct breakpoint within their respective groups (TETRA_2_, TETRA_7_; PARA_8_, PARA_6_; participant number in subscript). Notched boxplots are presented to compare the adjusted R^2^ values between the breakpoint and the no-breakpoint model for each participant **(C)**. Figures for all individual participants are provided in Supplementary Figure 3. V̇CO_2_, Carbon dioxide production; V̇E, minute ventilation; TETRA, wheelchair athlete with tetraplegia; PARA, wheelchair athlete with paraplegia; M, motor complete injury; S, sensory complete injury.

**FIGURE 3:**
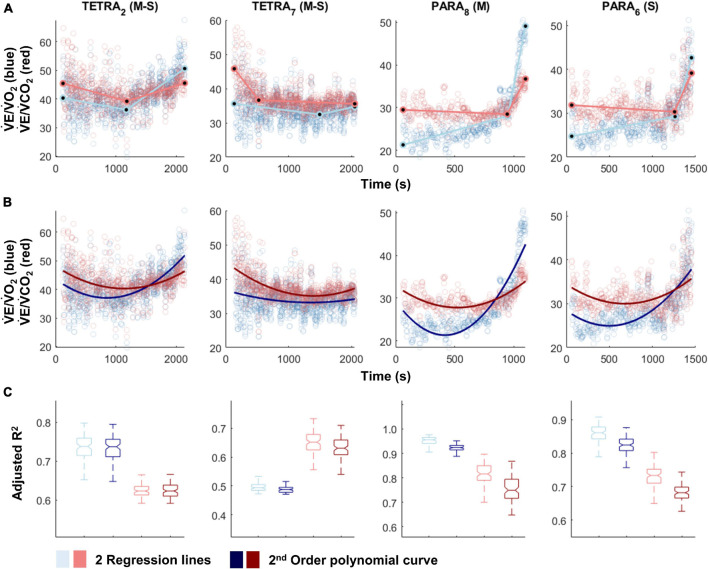
V̇E/V̇O_2_-time (blue) and V̇E/V̇CO_2_-time (red) plots fitted with the two linear regression lines (breakpoint model, ventilatory equivalent method; **A**) and a second order polynomial (no-breakpoint model; **B**) for two example athletes with tetraplegia and paraplegia (TETRA_2_, TETRA_7_; PARA_8_, PARA_6_; participant number in subscript). Notched boxplots are presented to compare the adjusted R^2^ values between the breakpoint and the no-breakpoint model for each participant **(C)**. Figures for all individual participants are provided in Supplementary Figure 4. V̇O_2,_ Oxygen uptake; V̇CO_2_, carbon dioxide production; TETRA, wheelchair athlete with tetraplegia; PARA, wheelchair athlete with paraplegia, M, motor complete injury; S, sensory complete injury.

**FIGURE 4:**
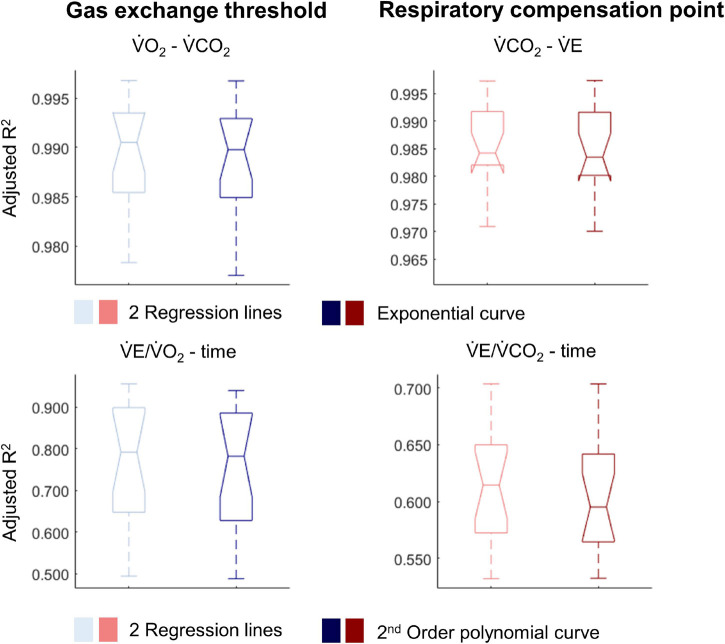
Notched boxplots to compare the adjusted R^2^ values of the breakpoint model (i.e., two linear regression lines) vs. the non-breakpoint model (i.e., exponential curve/second-order polynomial) to the respiratory data within the whole cohort of 17 wheelchair athletes. V̇O_2_, Oxygen uptake; V̇CO_2_, carbon dioxide production; V̇E, minute ventilation. Note that the notches extending beyond the 25^th^ or 75^th^ percentile indicate that the 95% CI interval ranges exceed the quartiles.

**FIGURE 5:**
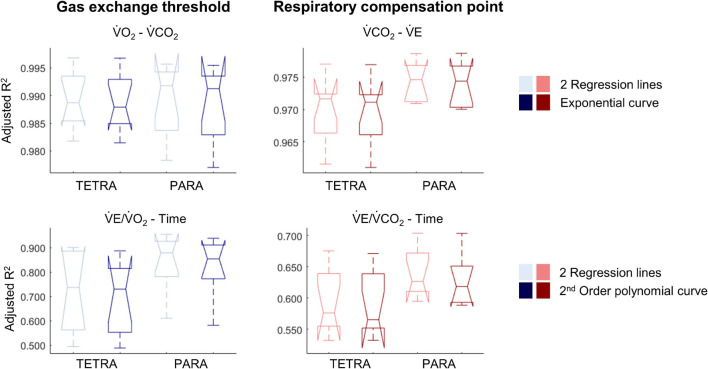
Notched boxplots to compare the adjusted R^2^ values of the breakpoint model (i.e., two linear regression lines) vs. the non-breakpoint model (i.e., exponential curve/second-order polynomial) to the respiratory data of 9 wheelchair athletes with a tetraplegia (TETRA) and 8 wheelchair athletes with a paraplegia (PARA). V̇O_2_, Oxygen uptake; V̇CO_2_, carbon dioxide production; V̇E, minute ventilation). Note that the notches extending beyond the 25^th^ or 75^th^ percentile indicate that the 95% CI interval ranges exceed the quartiles.

For most participants, there were overlaps in the adjusted R^2^ medians ± 95% CI ranges between breakpoint and no-breakpoint models fit to the V̇O_2_-V̇CO_2_ and V̇E/V̇O_2_-time data for the determination of the GET, as well as to the V̇E/V̇CO_2_-time and V̇CO_2_-V̇E data for the determination of the RCP (examples provided in Figures 1–3, all individual data provided in Supplementary Figures 2–4). For 10 of the 17 participants, the breakpoints identified by the two regression lines in the V̇E/V̇CO_2_-time data occurred before the ones identified in the V̇E/V̇O_2_-time data, i.e., the RCP occurred before the GET (Supplementary Figure 4). For the other participants (*N* = 7), these two breakpoints occurred at similar speeds (Δspeed range: 0.0–0.6 ms^–1^). On an overall group level, the adjusted R^2^ medians ± 95% CI interval ranges were not different between the breakpoint and the no-breakpoint models for the four approaches investigated (Figure 4). Within TETRA and PARA, there was an overlap in the 95% CI ranges between the breakpoint and the no-breakpoint models for either of the four approaches investigated (Figure 5). Despite a slight overlap in the 95% CI ranges between TETRA and PARA, the fit of both the breakpoint and no-breakpoint models was consistently lower in TETRA. The median model fit of all individual participants included in the current study is provided as Supplementary Excel File 1.

## Discussion

There was no difference between the fit of breakpoint vs no-breakpoint models for either of the ventilatory threshold (GET) or RCP approaches derived from data collected during a graded exercise test to exhaustion. This was the case both on an overall group level as well as within TETRA and PARA and indicates that these breakpoint models do not better explain the relationship of the data. Here we discuss two basic argumentations—either (1) breakpoints exist but the models employed are not sensitive enough to argue with confidence that they better explain the underlying data than no-breakpoint models, or (2) breakpoints do not exist.

### Breakpoints Exist, but the Models Employed Do Not Improve the Fit to the Data

Whilst the employed breakpoint models to determine the GET and RCP do not improve the goodness of fit to the data when compared with no-breakpoint models, breakpoints may occur after all. These breakpoints may be too subtle for the two regression lines to result in a better fit compared to the continuous no-breakpoint models. For example, breakpoints may be identified by only a handful of data points that do not follow a general curvilinear trend, too few to significantly alter any regression metrics. Automatic procedures may require improved fitting algorithms that more subtly address if functions, which best explain the data, are differentiable or not (the mathematical description of a breakpoint). Searching for more suitable differentiable continuous functions is only one option. Applying spectral density analysis procedures may be another. Machine learning, pattern recognition and artificial intelligence approaches may be a third. The success for any of such approaches depends heavily on the signal-variance ratio of the data, particularly if true breakpoints are subtle (as may be the case in this study).

In favor of the argument that breakpoints exist, GET and RCP methods are relatively widely used to identify intensity zones and, in some studies, high to very high inter- and intra-rater reliability has been reported (Gaskill et al., 2001; Amann et al., 2004; Pallarés et al., 2016; Au et al., 2018; Kouwijzer et al., 2019). There are few publications that use automatic determination of thresholds (Zignoli et al., 2019, 2020), with the majority opting for a fully subjective approach of visual determination (Aunola and Rusko, 1984; Gladden et al., 1985; Bhambhani et al., 1993; Holland et al., 1994; Kouwijzer et al., 2019). The inherent inaccuracy of this approach is reflected by a statement by Hopker et al. (2011), who noted that the GET and RCP methods “very crudely identify a time-point somewhere near to when the non-linearity in respiratory responses becomes more pronounced.” The investigated data set of the current study confirms this deviation from non-linearity, observed most strikingly in the ventilatory equivalent plots (Figure 3). However, our data also cast further doubt on the practical use of GET and RCP. Indeed, we showed that in the majority of cases, automatic threshold determination using the ventilatory equivalent method resulted in the RCP to be determined at *lower* exercise intensities than the GET, which is in contradiction to their definition, in which the RCP occurs at *higher* exercise intensity than the GET (Gaskill et al., 2001; Meyer et al., 2005). Given that the relationship in the V̇O_2_-V̇CO_2_ data is rather linear, it appears that it is mainly the rapid increase in V̇E that leads to the disproportionate rise in both the V̇E/V̇O_2_-time and the V̇E/V̇O_2_-time data. As such, it is not surprising that also for the remainder of cases, GET and RCP were determined in such proximity that their use in exercise prescription would be limited, i.e., it would not be possible to create a large enough moderate intensity zone within their boundaries. It is possible that subjective approaches are prone to subconscious bias (Hopker et al., 2011), leading to threshold identification that is “in line with underlying theory,” and/or being sufficiently far apart to allow creation of an intensity zone. Threshold identification based on traditional, subjective approaches may hence be limited to the *approximate* identification of a change in data patterns at best, or suffer from such bias at worst.

### Breakpoints Do Not Exist

Our findings support the general notion that respiratory changes in response to a gradual increase in exercise intensity are curvilinear. We here discuss this line of thought, especially as this may appear to be in contradiction to published research. Indeed, in contrast to the current study, distinct breakpoints in V̇E or related variables have been previously presented convincingly in figure format (Beaver et al., 1986; Wasserman, 1987). It is worth noting, however, that similar to the point made regarding bias in threshold identification, researchers have a tendency to present their “clearest dataset,” i.e., often the dataset with the least variability, and/or the one that matches the underlying theory (van der Steen et al., 2019). An example to support this notion in the present study are the data of PARA_8_ in Figure 1, which display one of the most distinct breakpoints in our study sample, even though the box plots show no significantly better fit of the breakpoint compared to the no-breakpoint model. To avoid such a selection bias, we here present *all* individual datasets (see Supplementary Figures 2–4) in addition to the data of example participants provided in Figures 1–3. These data indeed support the notion that V̇E is highly variable and increases in a curvilinear rather than a breakpoint fashion in response to an increase in exercise intensity. This pattern is likely reflected in V̇CO_2_ data, as it has previously been suggested that increases in V̇CO_2_ relate to increases in V̇E (Hopker et al., 2011). As V̇O_2_ and increases in submaximal exercise intensity are linearly related (Arts and Kuipers, 1994), it would hence not be expected that any relationships between exercise intensity, V̇O_2_, and/or V̇CO_2_ (derived) data should be characterized by any distinct breakpoints. Indeed, there is evidence that the transition from aerobic to anaerobic contributions with increasing exercise intensity occurs gradually, rather than at a specific intensity (Helal et al., 1987; Chamari and Padulo, 2015). Further, even though some studies report high intra-rater reliability as outlined above, there is also a considerable number of studies that report lower reliability (Shimizu et al., 1991; Bhambhani et al., 1993; Holland et al., 1994). This implies that the occurrence of a distinct point at which thresholds occur may either not be clear cut or, indeed, be non-existent. Altogether, this raises doubts on the concept of identifying breakpoints in respiratory data to distinguish low, moderate and high intensity exercise.

In addition, the absence of distinct breakpoints may be linked to the specific population tested. Breath-by-breath data are relatively variable, and when identified within a limited range of respiratory responses, breakpoints may not be visible. It is worth noting that respiratory responses are lower during upper-body compared to lower-body exercise, even in able-bodied participants (Nagle et al., 1984). Importantly, these responses are further reduced in people with more severe impairments such as a cervical SCI (Coutts et al., 1983), and accordingly, the ratio of the variability to the range of all respiratory variables was larger in TETRA in the present study (Supplementary Figures 2–4). This may explain to some extent that the goodness of fit of both, the breakpoint and no-breakpoint models, was lower in TETRA when compared to PARA, even though the fit between the breakpoint and no-breakpoint models was not significantly different between subgroups. Overall, we question how distinct these breakpoints are in the respiratory data of wheelchair athletes.

### Methodological Considerations

In the current study, we selected two very commonly used analytical methods to identify the GET and the RCP. There are other methods to identify the GET and RCP (Gaskill et al., 2001), and it remains speculative whether breakpoints are also not that distinct when applying these other methods. In addition to respiratory methods for identifying breakpoints that distinguish low, moderate and high intensity, a range of methods that set out to do the same are based on blood lactate concentration data (Faude et al., 2009). Using the concept of fitting breakpoint vs no-breakpoint curvilinear models to such data was beyond the scope of this study but is likely subject to the same inherent problems of absence of distinct breakpoints. Further, while the exponential or polynomial models have a strong grounding in existing literature (Fairshter et al., 1987; Dennis et al., 1992; Su et al., 2007), it remains to be investigated if there are curvilinear models with an even better fit to the data. A superior model fit of the curvilinear models may further strengthen our argument of the absence of distinct breakpoints in the respiratory data.

We focused on the respiratory responses of wheelchair court sport players with SCI and the interpretation of our results is therefore specific to this population. In intermittent sports, a greater endurance capacity is linked to better repeated sprint ability as well as faster recovery between games (McMahon and Wenger, 1998; Baumgart and Sandbakk, 2016), pointing out the relevance to determine endurance capacity markers such as GET or RCP in this cohort. Our results apply to highly trained athletes, who are characterized by a larger range of respiratory responses or markers of endurance performance than are seen in untrained wheelchair users (Janssen et al., 2002). Indeed, for untrained individuals, threshold determination may be further complicated by the lower range of respiratory variables. While we did not record information on indicators of reduced respiratory function, such as autonomic dysfunction and early onset sleep disordered breathing, the smaller range of the respiratory data of TETRA compared to PARA is in support of this.

Given that the present study was not designed to distinguish effects of disability and exercise mode, replicating it by including able-bodied upper-body trained participants would help to isolate the effect of the upper-body exercise mode from the disability. Finally, future investigations are needed to look into whether our findings of subtle or absent breakpoints in the respiratory responses during upper-body exercise extend to lower- and whole-body exercise, which elicit higher and less variable respiratory responses (Sawka et al., 1982; Castro et al., 2011).

### Practical Applications

We here show that breakpoints in the respiratory data of wheelchair athletes obtained during upper-body exercise testing are either very subtle, or they do not exist. This information is of value to both athletes and coaches, as it indicates that the use of the methods employed in the current study should be discouraged to identify low-, moderate- and high-intensity zones. As this implies that intensity zone boundaries cannot be accurately determined based on ventilatory data from graded exercise tests to exhaustion, such intensity zones need to be verified using alternative methods. This may include performing steady state exercise tests to establish the maximum lactate steady state or the highest exercise intensity that does not raise blood lactate above resting concentrations (“the lactate threshold”) (Beneke et al., 2011). Alternatively, critical power may be determined using a series of supramaximal sprints (Jones et al., 2010). If verified as such, thresholds may remain a valuable tool to guide training. If applicable, training zones should be assessed separately for different exercise modalities.

## Conclusion

The goodness of fit between breakpoint and no-breakpoint models did not differ for either of the ventilatory or respiratory compensation threshold approaches investigated in this study. This held true on an overall group level as well as within TETRA and PARA, indicating that breakpoint models do not best explain the relationship of respiratory data collected during a graded upper-body exercise test to exhaustion. Two lines of reasoning may explain these findings: (1) breakpoints in these respiratory data do not exist, as has been argued previously; (2) breakpoints exist, but are too subtle to result in a significant difference in fit between the investigated breakpoint and no-breakpoint models. To conclude with more certainty on the respective influences of (upper-body) exercise modality or disability (e.g., respiratory, muscular, autonomic limitations) on our findings, future investigations should include able-bodied, upper-body trained participants.

## Data Availability Statement

The data analyzed in this study is subject to the following licenses/restrictions: While the raw data is not publicly available, the median model fit of all individual participants included in the current study is provided in a supplement (Supplementary Material). Requests to access these datasets should be directed to JKB, julia.k.baumgart@ntnu.no.

## Ethics Statement

The studies involving human participants were reviewed and approved by the Ethical Committee of Loughborough University. The participants provided their written informed consent to participate in this study.

## Author Contributions

JKB, GE, KG, VG-T, and CL: conceptualization, methodology, and writing—review and editing. JKB, GE, and CL: formal analysis and investigation. JKB: writing—original draft preparation. All authors contributed to the article and approved the submitted version.

## Conflict of Interest

The authors declare that the research was conducted in the absence of any commercial or financial relationships that could be construed as a potential conflict of interest.

## Publisher’s Note

All claims expressed in this article are solely those of the authors and do not necessarily represent those of their affiliated organizations, or those of the publisher, the editors and the reviewers. Any product that may be evaluated in this article, or claim that may be made by its manufacturer, is not guaranteed or endorsed by the publisher.
