# Supplementary figures and images for: A Reappraisal of Ventilatory Thresholds in Wheelchair Athletes With a Spinal Cord Injury: Do They Really Exist?
Source: Front Physiol. 2021 Nov 26;12:719341. doi: 10.3389/fphys.2021.719341 (PMC8664409; doi:10.3389/fphys.2021.719341)

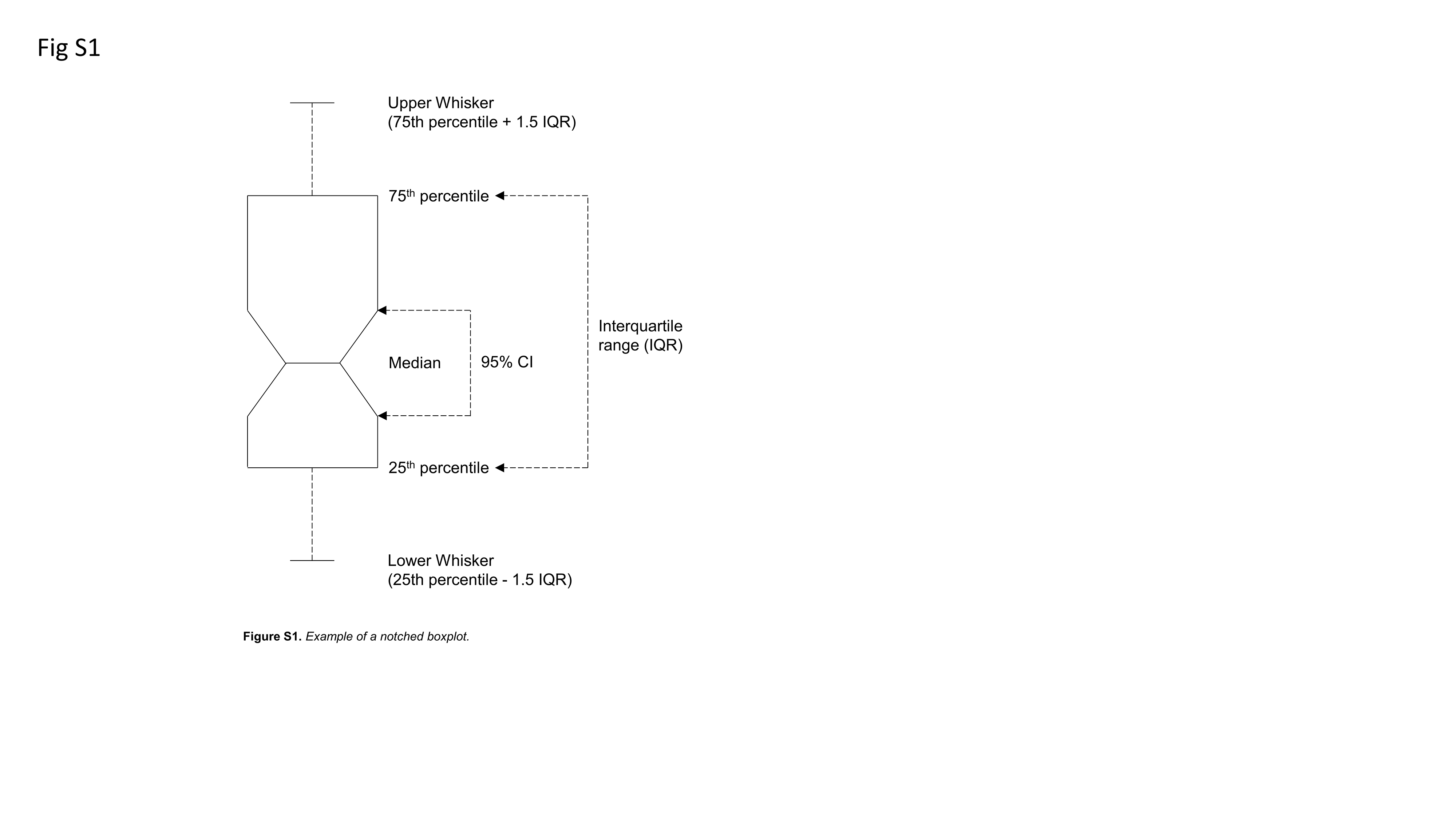

Supplement: Supplementary file 1 [file Image_1.tif]

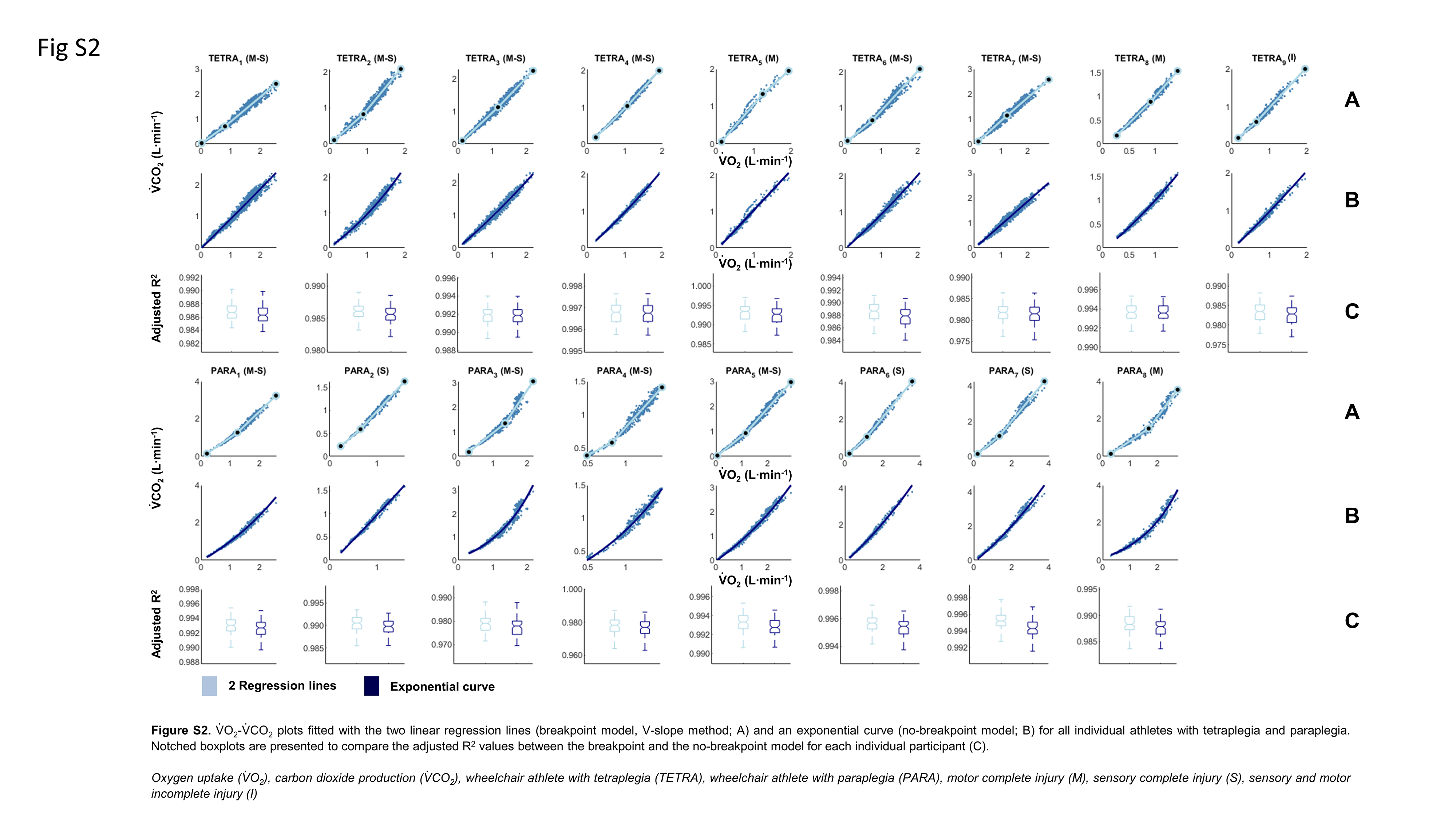

Supplement: Supplementary file 2 [file Image_2.tif]

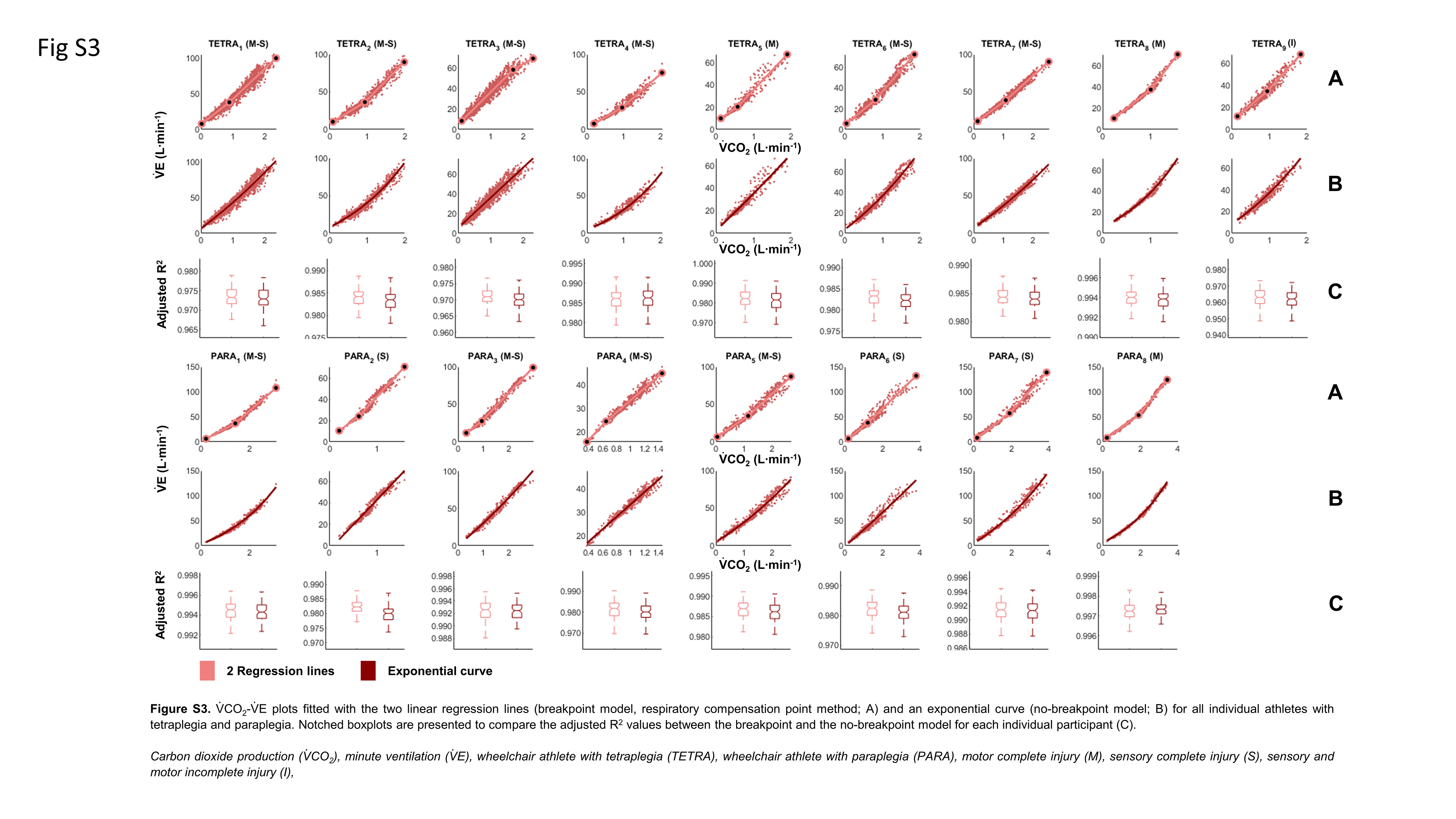

Supplement: Supplementary file 3 [file Image_3.tif]

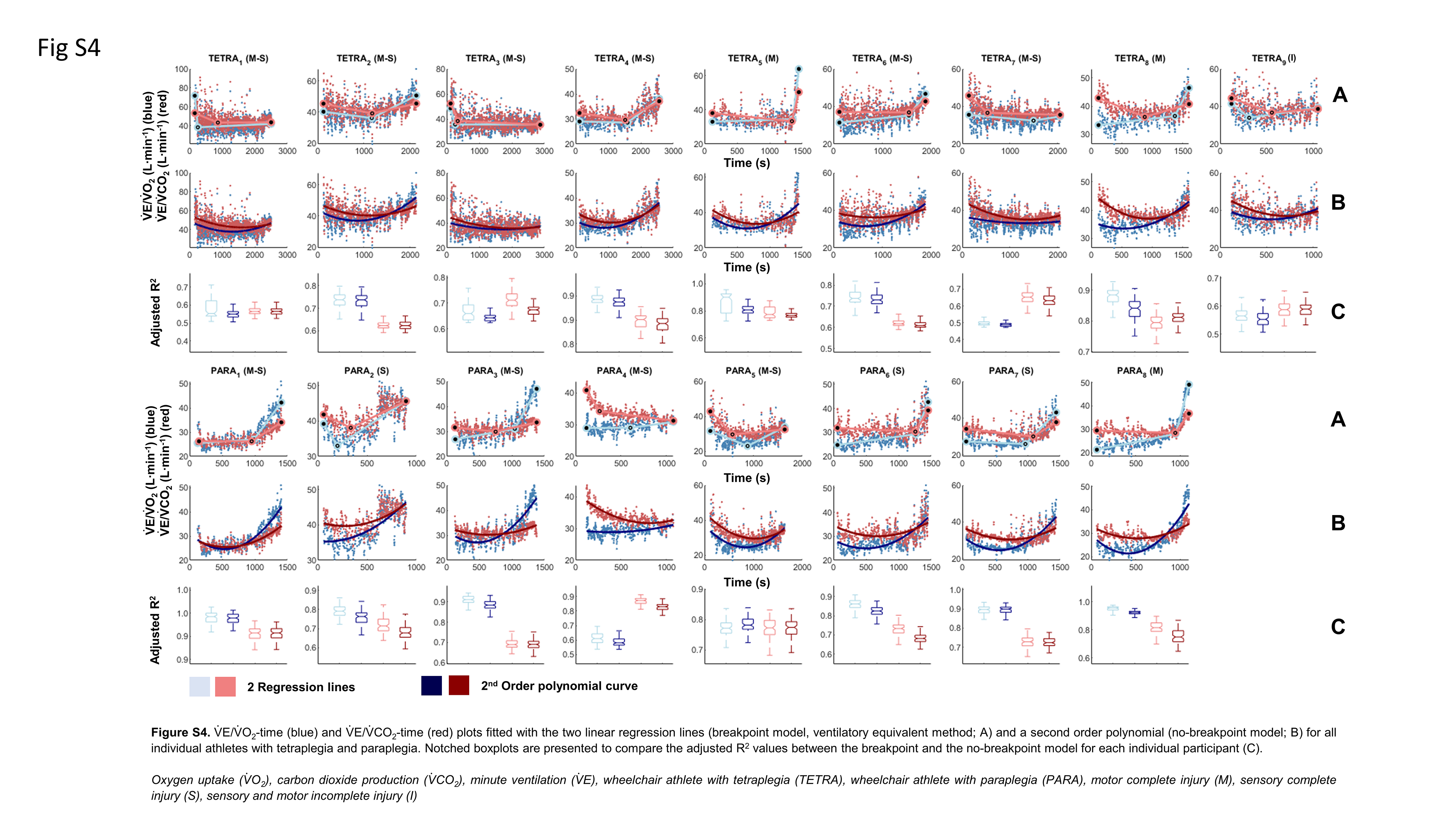

Supplement: Supplementary file 4 [file Image_4.tif]
